# Adjusting to Your Surroundings: An Inquiry-Based Learning Module to Teach Principles of Mechanobiology for Regenerative Medicine

**DOI:** 10.1007/s43683-023-00130-6

**Published:** 2023-12-04

**Authors:** Christopher J. Panebianco, Madhura P. Nijsure, Erin E. Berlew, Annie L. Jeong, Joel D. Boerckel

**Affiliations:** 1 Department of Orthopaedic Surgery, University of Pennsylvania, 3450 Hamilton Walk, 371 Stemmler Hall, Philadelphia, PA 19104, USA; 2 Center for Engineering Mechanobiology, University of Pennsylvania, Philadelphia, PA, USA

**Keywords:** Inquiry-based learning, Hands-on experiment, Engineering education, Mechanobiology, Biomaterials, Regenerative medicine, Cell–biomaterial interactions

## Abstract

Mechanobiology is an interdisciplinary field that aims to understand how physical forces impact biological systems. Enhancing our knowledge of mechanobiology has become increasingly important for understanding human disease and developing novel therapeutics. There is a societal need to teach diverse students principles of mechanobiology so that we may collectively expand our knowledge of this subject and apply new principles to improving human health. Toward this goal, we designed, implemented, and evaluated a hands-on, inquiry-based learning (IBL) module to teach students principles of cell–biomaterial interactions. This module was designed to be hosted in two 3-h sessions, over two consecutive days. During this time, students learned how to synthesize and mechanically test biomaterials, culture bacteria cells, and assess effects of matrix stiffness on bacteria cell proliferation. Among the 73 students who registered to participate in our IBL mechanobiology module, 40 students completed both days and participated in this study. A vast majority of the participants were considered underrepresented minority (URM) students based on race/ethnicity. Using pre/post-tests, we found that students experienced significant learning gains of 33 percentage points from completing our IBL mechanobiology module. In addition to gaining knowledge of mechanobiology, validated pre/post-surveys showed that students also experienced significant improvements in scientific literacy. Instructors may use this module as described, increase the complexity for an undergraduate classroom assignment, or make the module less complex for K-12 outreach. As presented, this IBL mechanobiology module effectively teaches diverse students principles of mechanobiology and scientific inquiry. Deploying this module, and similar IBL modules, may help advance the next generation of mechanobiologists.

## Introduction

### Mechanobiology in Regenerative Medicine

Mechanobiology is an interdisciplinary field that aims to understand how physical forces impact biological systems. Elucidating the mechanisms by which mechanical properties of a cell’s surrounding environment can impact cellular responses is important because many human pathologies are associated with altered tissue stiffness. For example, tissue-level changes in mechanical properties can trigger a pathogenic cellular response, and the inability of a cell to respond to mechanical stimuli can trigger disease [1]. On the other hand, appropriate mechanical stimulation can promote repair and regeneration [2]. Developing a deeper understanding of mechanobiology in disease has led to the development of mechanobiology-inspired therapies for numerous tissues, including bone [3], muscle [4], tendon [5], skin [6], heart [7], and liver [8]. To continue expanding the boundaries of mechanobiology research, there is a need to educate diverse students about mechanobiology.

Hands-on education modules are an effective way of teaching principles of bioengineering and mechanobiology [9], and using inexpensive materials to execute these modules ensures they are more accessible to diverse trainees. There are numerous published undergraduate bioengineering modules, which cover aspects of mechanobiology; however, there are few accessible modules that enable students to explore mechanobiology. For example, educators have developed hands-on modules to teach students about mechanically testing biomaterials [10–13], tissue-level mechanics [14–16], and cell-level mechanics [17, 18]. While these modules effectively cover mechanobiology topics, they require expensive reagents, sophisticated testing equipment, and the ability to safely culture mammalian cells. Primarily undergraduate institutions (PUIs) and community college, which have high populations of underrepresented minority (URM) students, generally do not have the resources to execute such expensive modules. Thus, there is a need to develop more accessible modules, which are inexpensive and easily deployable, to teach diverse undergraduates about mechanobiology.

Interactions between cells and the substrate on which they are cultured are extremely important for designing regenerative strategies. In a seminal paper by Engler *et al*., researchers found that matrix elasticity can direct stem cell lineage specification [19]. This discovery introduced the biophysical environment as a design variable that can be manipulated, which has enhanced the field of functional tissue engineering [20]. To the authors’ knowledge, Saterbak *et al*. were the only group to publish an educational module explicitly covering the topic of cell–biomaterial interactions. In this module, students characterized the chemical properties of poly(l-lactic acid) and poly(dl-lactic-*co*-glycolic acid) films, then assessed how chemical properties impacted the attachment and proliferation of human dermal fibroblast cells [21]. While this module nicely demonstrates how a cells chemical environment affects its phenotype, it does not teach students the importance of a cell’s physical environment. This lack of published educational modules centered on mechanobiology represents a major gap in the field of mechanobiology education, which may limit student access to the field of regenerative mechanobiology and advances made in the field.

### Pedagogical Framework

We designed this mechanobiology module using an inquiry-based learning (IBL) approach, because of the benefits IBL has over traditional learning. In a traditional learning approach, instructors lecture students to teach information [22], then students take exams to demonstrate their knowledge [23, 24]. In a laboratory classroom setting, traditional learning implies that students are only responsible for collecting and analyzing data. There are numerous critiques of traditional learning, including creating student–teacher power imbalances, producing non-active learners, and promoting superficial learning [25, 26]. Several non-traditional approaches exist to combat the limitations of traditional learning, including active learning [27], entrepreneurial mindset learning [28], and IBL [26]. We chose to design this mechanobiology module using an IBL framework.

In an IBL approach, students discuss the material with their peers and teachers while they are learning, cite internal and external scientific evidence, and present their findings through written work and/or oral presentations [29, 30]. In a classroom setting, the key features of IBL are planning, retrieving, processing, creating, sharing, and evaluating. The initial planning phase involves finding information and figuring out general questions, while the retrieving phase involves focusing on a particular topic. Next, students process the information into a thesis and create an argument to share with their peers. After sharing, students evaluate their learning [31]. Similar principles distinguish an IBL laboratory class from a traditional laboratory class. In addition to collecting and analyzing data, IBL laboratory classes challenge students to make observations, pose research questions, design experiments, repeat their experiments, and report their findings [32].

The merits of IBL have been demonstrated in several large-scale studies. One of the first longitudinal studies showed that middle school children taught with an IBL approach had greater learning outcomes than students who participated in traditional learning [33]. The efficacy of IBL was shown at an even larger scale through the Promoting Inquiry in Mathematics and Science Education Across Europe (PRIMAS) Project. This project, which was conducted across 14 primary and secondary institutions across 12 European countries, demonstrated that IBL fostered superior learner competence to traditional learning techniques [34]. In addition to the general benefits of IBL over traditional learning approaches, implementing IBL in the laboratory classroom setting has been shown to support better student outcomes for URM students [35, 36]. Given extant critiques of traditional learning, and the published benefits of IBL, we chose an IBL approach to design our mechanobiology module on cell–biomaterial interactions.

### Module Design, Implementation, and Evaluation

This study designed, implemented, and evaluated an IBL mechanobiology module to teach students about cell–biomaterial interactions. This IBL mechanobiology module was designed to be hosted over two consecutive days, with a 3-h session each day. During this time, students learned how to synthesize and mechanically test biomaterials, culture bacteria cells, and modulate cell phenotype through biophysical stimuli. We deployed this IBL mechanobiology module at PUIs and community colleges that have high populations of students who are considered underrepresented in science, technology, engineering, and mathematics (STEM) [37]. Focusing outreach efforts on underrepresented minority (URM) students was considered a priority since these efforts can help diversify STEM fields [38–40]. Since these categories of institutions have more limited resources relative to large research institutions, we designed our IBL mechanobiology module to use inexpensive materials (i.e., agar and *Escherichia coli*) and eliminate the need for expensive equipment (i.e., mechanical testing devices and biosafety cabinets). This design allowed us to rigorously teach principles of mechanobiology, while keeping the module accessible to diverse students from a broad range of institutions.

This IBL mechanobiology module was evaluated to determine if it (1) taught students principles of mechanobiology and (2) enhanced scientific literacy. Learning gains were assessed using pre/post-tests and scientific literacy was assessed using validated pre/post-surveys. Overall, the goal was for this module to promote positive learning gains and enhance scientific literacy for diverse undergraduate students with limited knowledge of mechanobiology.

## Materials and Methods

### Module Overview

**Fig. 1:**
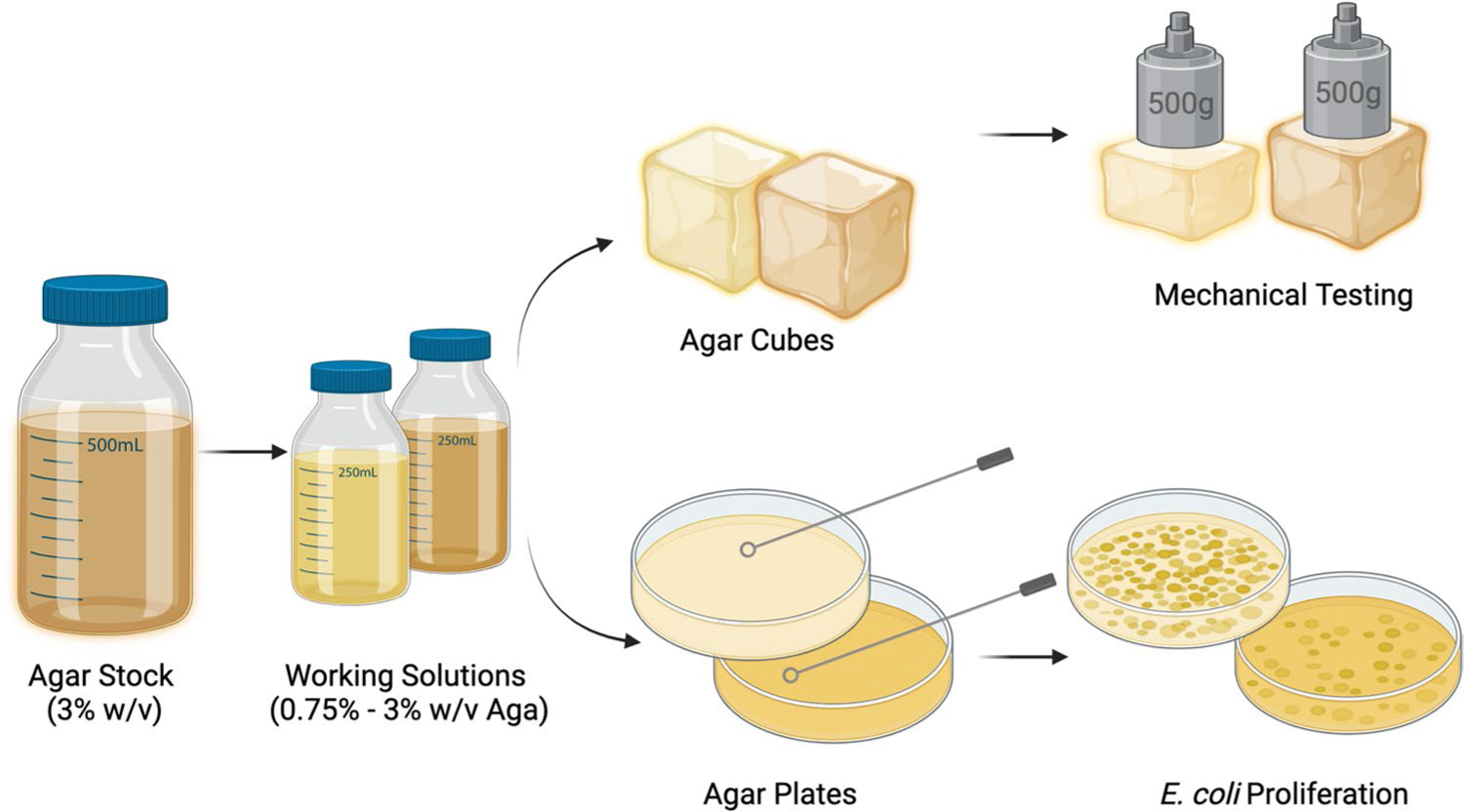
Schematic representation of inquiry-based learning (IBL) mechanobiology module. Students were provided with a 3% (w/v) agar stock solution, then asked to make two working solutions from 0.75 to 3%. Using these two concentrations, students cast agar cubes for mechanical testing and agar plates for *E. coli* culture

The IBL mechanobiology module was designed to be hosted over two consecutive days, with a 3-h session each day. The module can be divided into three stages: (1) pre-activity exercises, (2) IBL activity, and (3) post-activity exercises (Table 1). During the first session, students cast agar hydrogel cubes and agar plates with different macromer concentrations of agar, mechanically tested hydrogel cubes, and cultured *E. coli* on agar plates. During the second session, students analyzed their mechanical data and qualitatively assessed how substrate stiffness impacts *E. coli* proliferation (Fig. 1). In accordance with inquiry-based learning (IBL) pedagogy, students worked collaboratively to generate hypotheses, conduct experiments, and analyze their results, with instructor guidance.

### Materials and Solution Preparation

The IBL mechanobiology module was conducted in laboratory spaces that were equipped with gloves, autoclavable bottles, micropipettes, micropipette tips, pipette aids, serological pipettes, lab markers, and lab tape. Additionally, the laboratory spaces had access to a refrigerator/cold room and autoclave. Importantly, autoclave access is not necessary for implementation of this module, as agar solutions can be prepared using a standard microwave oven. The following additional materials were purchased to complete this IBL mechanobiology module:

- Agar powder (Thomas Scientific, Swedesboro, NJ, Product Number: C752V97),
- LB broth (Thomas Scientific, Product Number: C833T86),
- Silicone 2″ ice cube tray (Amazon, Seattle, WA, Product Number: B00395FHRO),
- 5 kg calibration weight (Amazon, Product Number: B08R8MNMQF),
- 2 kg + 1 kg calibration weight set (Amazon, Product Number: B0C4Z2DZJC),
- 10–500 g calibration weight set (Amazon, Product Number: B01EIHQP1I),
- Competent *E. coli* cells (New England Biolabs, Ispwich, MA, Product Number: C2984H),
- Polystyrene bacteria culture tubes (Thomas Scientific, Product Number: 1227Z60),
- Disposable inoculation loops (Thomas Scientific, Product Number: 1233L17),
- 10 cm Petri dishes (Thomas Scientific, Product Number: 1188N81),
- Freezer gallon bags (Amazon, Product Number: B093WPZF1Y).

One day prior to the activity, instructors prepared a bacteria culture by scraping a P200 pipette tip on the frozen stock of competent *E. coli* cells, placing the tip in a bacteria culture tube containing 5 mL of LB broth, and incubating the culture at 37°C overnight with agitation at 250 rpm. This method yields a highly confluent stock bacteria culture, which should be diluted. Instructors found that diluting the stock by 1:1,000,000 with LB broth at the start of the activity yielded a working bacteria solution that produced distinct colonies after homogenous streaking along the bacteria plate. If using this method, instructors should prepare 1 mL of the working bacteria solution for each group. Instructors with more experience culturing bacteria may choose not to dilute the stock bacteria culture and teach students more sophisticated streaking methods (e.g., quadrant streaking procedure).

In addition to the bacteria solutions, instructors also prepared one solution containing 3% (w/v) agar and 2.5% LB broth in DI water, and a separate 2.5% LB broth solution (500 mL of each solution per student). LB broth was included in the 3% agar stock solution to reduce the effects of nutrient concentration confounding bacteria growth results. The additional 2.5% LB broth solution was prepared for diluting the 3% agar stock solution. All solutions were autoclaved for sterility and to completely dissolve powders in solution. After autoclaving, solutions were kept in the autoclave or placed in a 60°C water bath because agar will harden at room temperature. Of note, the 3% agar stock solution requires sufficient headspace when being autoclaved to prevent overflow of the solution. Therefore, the 500 mL 3% agar and 2.5% LB broth solution were prepared in a 1-L autoclavable bottle. If instructors do not have access to an autoclave, they may dissolve solutions using a microwave oven. The instructor team leading this module was able to successfully dissolve agar in solution using a microwave, if students ran out of agar.

### Institution Information and Student Activity Groups

Students participating in our IBL mechanobiology module were enrolled at the Community College of Philadelphia (CCP), the University of Puerto Rico (UPR) - Cayey, and UPR - Mayaguez. 53 undergraduate students participated in at least 1 day of our IBL mechanobiology module. This included 15 from CCP, 14 students from UPR - Cayey, and 24 students from UPR - Mayaguez. Of these 53 students, 40 students completed both days of the activity, provided informed consent for the study, and voluntarily completed our pre/post-test assessments. CCP students were recruited from those enrolled in the Science and Technology degree programs, and students from UPR - Cayey and [UPR - Mayaguez were recruited from those enrolled in the National Institutes of Health (NIH) Research Training Initiative for Student Enhancement (RISE) Program at their institutions. At all institutions, students were allowed to choose their own activity groups of 3–4. All questions were approved by the University of Pennsylvania Institutional Review Board (IRB, IRB Protocol # 852812). Data was only analyzed for students who completed an informed consent form.

### Pre-Activity Lecture and Exercise

During the pre-activity exercise, instructors provided a brief background on mechanobiology, biomaterials and cell–biomaterial interactions. We used active learning strategies throughout this portion of the pre-activity exercise (e.g., think-pair-share, multiple hands/voices) [41] because this pedagogy has been shown to improve student performance in science, technology, engineering, and mathematics (STEM) courses [42]. Moreover, we employed active learning strategies because a majority of our cohort identified as underrepresented minorities (URM) and active learning helps narrow achievement gaps for students from underrepresented groups [27, 43]. After the lecture, students formed their activity groups and began discussing independent variables they would be manipulating (e.g., agar concentration), their mechanical testing scheme, quantitative and qualitative output measurements, and specific hypotheses they would test. Incorporating additional degrees of inquiry implementation, beyond merely collecting and analyzing data, is essential for the IBL philosophy [32]. These student-led discussions with instructor guidance ensured all group members had an appropriate level of background knowledge to conduct this IBL mechanobiology module and were comfortable using IBL techniques [44]. We chose this approach assuming that students had no previous experiences with IBL prior to the module.

### Agar Cube Generation and Mechanical Testing Procedure

**Fig. 2:**
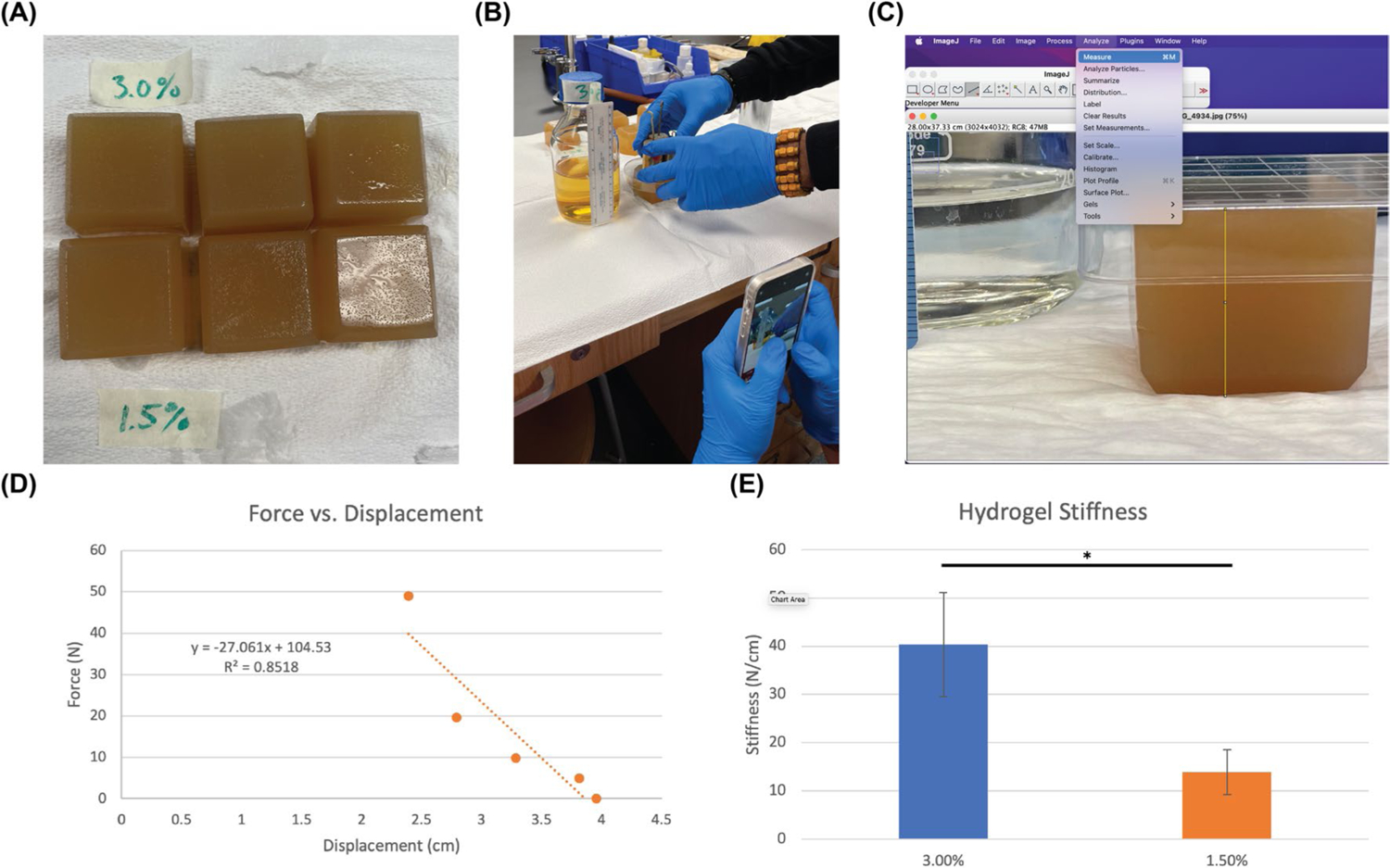
Student-generated data demonstrating successful agar cube generation and compression testing. **A** Representative agar cubes with 3% (w/v) and 1.5% agar. **B** Manual mechanical testing scheme. Of note, students included a ruler and label in each picture, and took pictures parallel to the mechanical testing plane. **C** ImageJ measurement of agar cube height. **D** Representative student-generated force–displacement graph for an individual agar cube. **E** Compiled student-generated stiffness data with statistics showing the effects of agar concentration on cube stiffness

The activity portion of the IBL mechanobiology module was student-led. Instructors were facilitators of the hands-on activity and answered questions, but students conducted experiments and recorded output measurements. In the first portion of the activity, students tested how the concentration of agar in pre-crosslinked polymer solutions would impact the compressive properties of resultant physically entangled hydrogels. To do this, we adapted a previously described method for students to conduct at-home compressive testing of gelatin hydrogels [12]. First, students calculated the volumes of agar and LB broth required to make 100 mL hydrogel cubes with 0.75–3% agar. This concentration range was provided by instructors based on previous experimentation with the material; however, in accordance with IBL, students had the freedom to choose any two concentrations within this range. Next, students mixed the agar and LB broth solutions in the ice cube trays to create hydrogel cubes of the desired concentration. Of note, solutions were kept warm in the autoclave or a 60°C water bath prior to mixing, and LB broth was added to molds first to ensure that the agar did not harden prior to mixing with the LB broth diluent. Students were instructed to generate three replicates for each condition (*n*=3) to allow statistical analysis. Poured ice cube trays were incubated in a refrigerator or cold room at 4°C for approximately 30 min prior to mechanical testing (Fig. 2A).

Once the hydrogel cubes set in the fridge, students implemented their mechanical testing scheme to calculate the compressive stiffness of their hydrogels. Similar to how students used IBL to pick their agar concentrations, students were challenged to design their own mechanical testing schemes. Generally, this consisted of placing one side of a petri dish on top of the cube, then using a smartphone camera to take pictures of the hydrogel with different calibration weights on top of it (Fig. 2B). Students were instructed to add weights from lightest to heaviest to simulate automated mechanical testing and to avoid permanent deformations to the hydrogel, which would lead to confounding results with lower weights. Testing images were taken parallel to the testing plane and included a ruler in frame to measure displacement with ImageJ (NIH, Bethesda, MD) (Fig. 2C). ImageJ was chosen because it is a free, open-source software that is commonly used for biomedical research. The resulting force–displacement data from ImageJ was recorded in Microsoft Excel (Microsoft, Redmond, WA) or Google Sheets (Google, Mountain View, CA). Students used one of these programs to calculate the stiffness of each hydrogel (Fig. 2D). Using Excel or Google Sheets, students conducted a Student’s *t*-test to find significant differences between their hydrogels (Fig. 2E). Ultimately, students found that increasing the concentration of agar would increase the compressive stiffness of resultant hydrogel cubes.

### Agar Plate Generation and Bacteria Culture Procedure

**Fig. 3:**
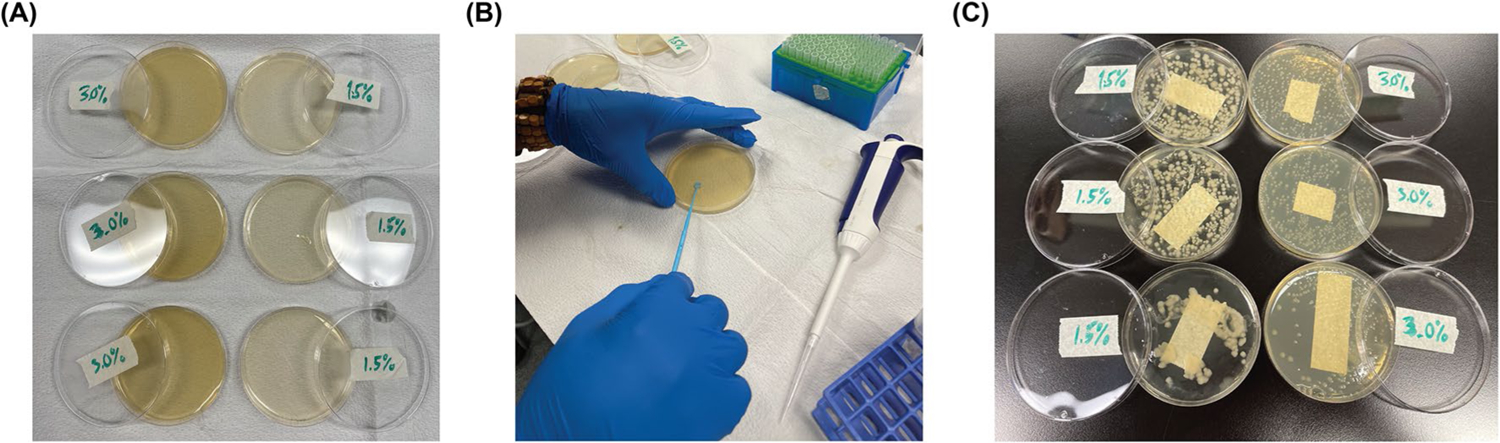
Student-generated data demonstrating successful agar plate generation and *E. coli* culture. **A** Representative agar plates with 3% (w/v) and 1.5% agar. **B** Students streaking bacteria along the entire surface of an agar plate. **C** Representative images of *E. coli* cultured overnight on 1.5% (soft) and 3% (stiff) agar plates showing the effect of substrate stiffness on *E. coli* proliferation

In the second portion of the activity, students tested how substrate stiffness would impact the proliferation of *E. coli* bacteria. This activity was inspired by a paper from Saha *et al*., which determined that *E. coli* exhibited more rapid growth on softer films compared to stiffer films [45]. First, students calculated the volumes of agar and LB broth required to make 20 mL agar plates with the concentrations they chose for their mechanical testing (2.5 agar cube generation and mechanical testing procedure). Next, students mixed the agar and LB broth into Petri dishes to create agar plates with the desired concentration. Of note, solutions were kept warm in the autoclave or a 60°C water bath prior to mixing, and LB broth was added to molds first to ensure that the agar did not harden prior to mixing with the LB broth diluent. Agar plates were also swirled immediately after adding agar to ensure complete mixing of the agar and LB broth solutions. Students were instructed to generate three replicates for each condition (*n*=3) to allow for statistical analysis. Poured agar plates were incubated in a refrigerator or cold room for approximately 10 min prior to culturing bacteria (Fig. 3A).

Once the agar plates set in the fridge, students cultured bacteria on their agar plates with varied stiffness. Students pipetted 100 μL of the working *E. coli* solution (2.2 Materials and solution preparation) onto each agar plate, then used a sterile inoculation loop to spread the solution over the entire surface of the agar plate. Students were advised to spread bacteria on the stiffer substrates first because they could learn the technique with less risk of puncturing the agar plate surface of the softer substrates (Fig. 3B). After briefly drying (<5 min), bacteria plates were placed upside down (i.e., agar side up) in a freezer gallon bag and incubated overnight at 37 °C without agitation. The following day, students qualitatively assessed how substrate stiffness impacted *E. coli* proliferation and discussed how they could use ImageJ to quantify their findings (e.g., count colonies, measure colony diameter). Ultimately, students found that *E. coli* proliferated more quickly on softer substrates (i.e., agar plates with lower concentrations of agar) (Fig. 3C).

### Post-Activity Exercise

After completing the activity, students were guided through a post-activity exercise and discussion. Similar to the pre-activity exercise, the first portion of the post-activity exercise was student-led. Students were challenged to summarize their findings with appropriate statistical analysis, critically analyze the limitations of their experimental approach, and discuss their findings in the context of real-world applications. Incorporating these degrees of inquiry into the IBL module distinguish our module from a traditional laboratory module [32]. In the second portion of the post-activity exercise, instructors used active learning strategies to teach students about mechanically regulated signal transduction, follow-up molecular analyses, and published applications of mechanobiology in regenerative medicine.

### Learning Module Evaluation

Students were given pre- and post-tests to evaluate the learning gains from completing this IBL mechanobiology module. The pre/post-tests, which were composed by the authors, consisted of five multiple choice test questions (TQs) about the mechanical properties of biomaterials, cell–biomaterial interactions, and applications of mechanobiology in tissue degeneration/regeneration. This pre/post-test length was chosen so that it could assess comprehension of multiple learning objectives without being repetitive and placing extra burden on students participating in this voluntary study. Furthermore, assessments of a similar length have been used to assess the learning gains resulting from similar biomedical engineering education modules [10, 12, 13, 46]. Pre-tests were administered on the first day of the module, prior to the start of the pre-activity exercise. Post-tests were administered on the second day of the module, after the post-activity exercise. Students were not allowed to review their pre-test prior to taking the post-test to avoid students simply recalling correct answers. Data was only analyzed for students who provided informed consent and completed both tests.

Students were also given pre- and post-surveys to assess changes in their scientific literacy as a result of completing this IBL mechanobiology module. The pre/post-surveys used in this module were a subset of those from the Scientific Literacy and Student Value in Inquiry-guided Lab Survey (SLIGS) [47]. Students answered questions on a Likert Scale, and each response was assigned a numerical value to allow for statistical analysis (i.e., “Not Confident” = 0, “Somewhat Not Confident” = 1, “Somewhat Confident” = 2, and “Confident” = 3).

Pre/post-tests and pre/post-surveys were distributed using Qualtrics (Qualtrics, Seattle, WA), which required students to provide informed consent prior to answering questions. To ensure student anonymity, the form asked students to provide an ID number, rather than their name. The ID number consisted of the initials of their institution and the last 4 digits of their phone number (e.g., CCP7762) for CCP. Statistical analysis was conducted using GraphPad Prism^®^ software version 9 (GraphPad Software, San Diego, CA). Average scores of pre/post-tests and pre/post-surveys were compared using paired Student’s *t*-tests to determine significant learning gains and significant improvements in scientific literacy (*α* = 0.05).

## Results

### Student Demographic Information

**Fig. 4:**
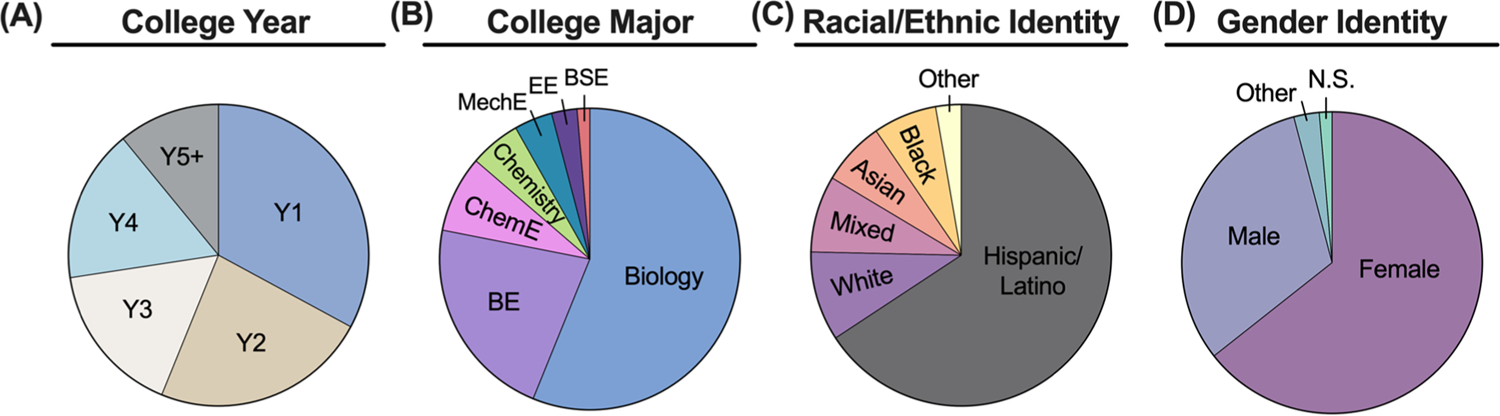
Demographic information of students who registered for the IBL mechanobiology module. **A** Breakdown of college year. **B** Breakdown of college major. *BE* bioengineering, *ChemE* chemical engineering, *MechE* mechanical engineering, *EE* electrical engineering, *BSE* engineering science. **C** Breakdown of racial/ethnic identity. **D** Breakdown of gender identity. *NS* non-specified gender identity

Our inquiry-based learning (IBL) mechanobiology module was deployed at CCP, UPR - Cayey, and UPR - Mayaguez. Across all universities, 73 students registered to complete the module. Most students were in either the first or second year of their undergraduate program (Fig. 4A). Approximately 75% of these students were majoring in biology or bioengineering, and the remaining students were majoring in chemistry or another engineering discipline (Fig. 4B). Greater than 80% of the students identified as Hispanic/Latino, black/African American, mixed race, or other; thus, a vast majority of our cohort consisted of individuals who are underrepresented in STEM based on race/ethnicity (Fig. 4C). Additionally, most of the students did not identify as male, indicating that most students in our cohort were gender minorities in STEM (Fig. 4D). Recruiting racial/ethnic and gender minorities to participate in our module was done purposefully, since focusing outreach efforts on individuals who are traditionally underrepresented in STEM based on race, ethnicity, gender, sexual orientation, socioeconomic status, etc., is especially important for diversifying STEM fields [38–40].

### Pre/Post-Test Assessment

**Fig. 5:**
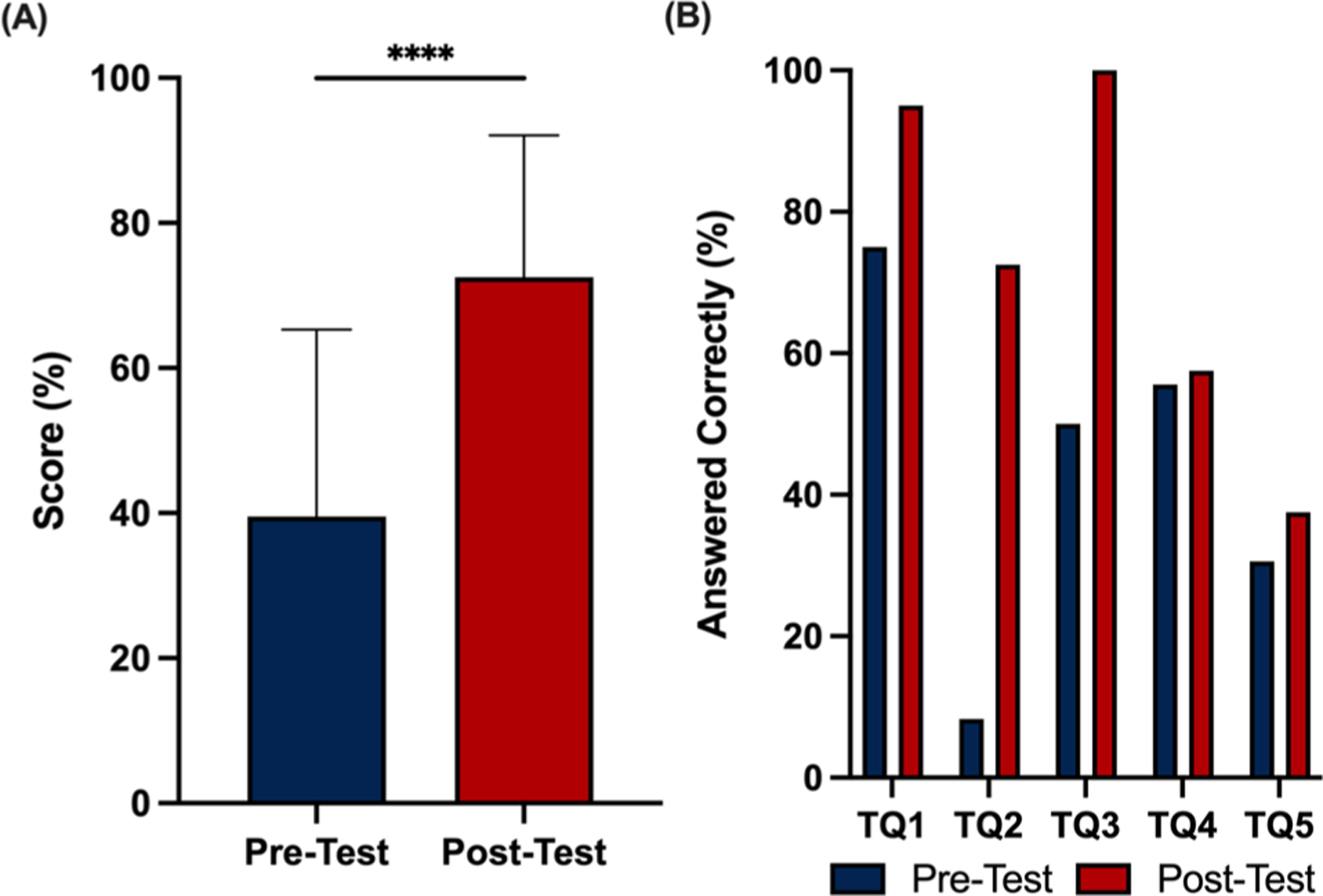
Students showed significant learning gains by pre/post-test. **A** Average student pre/post-test score. **B** Percentage of students who answered individual questions correctly on pre/post-tests. ****p<0.0001 using paired Student’s *t*-test

Across all three institutions, forty students (*N*=40) participated in the pre/post-test assessment. Multiple choice test questions (TQs) were written by instructors to test student understanding of biomaterial synthesis, mechanical testing of biomaterials, cell–biomaterial interactions, and applications of mechanobiology in studying degeneration/regeneration (“Appendix 1”). Results of our pre/post-test assessment showed that students experienced significant learning gains from participating in our IBL mechanobiology module. Specifically, the average score increased from 39.5% on the pre-test to 72.5% on the post-test (Fig. 5A).

In addition to significant increases in overall score, we found that the percentage of students who answered each TQ correctly was greater in the post-test than the pre-test (Fig. 5B). TQ1, TQ2, TQ3, and TQ4 were all lower level questions by Bloom’s Taxonomy [48], designed to have students recall facts. The percentage of students who answered TQ1, TQ2, and TQ3 substantially increased from the pre-test and the post-test because these questions tested direct concepts IBL mechanobiology module: mechanically testing biomaterials, biomaterial synthesis, and cell–biomaterial interactions. On the contrary, there was only a modest increase in the percentage of students who answered TQ4 correct in the post-test compared to the pre-test. This was expected because the concept of how mechanical cues regulate signal transduction was not directly tested in the IBL module. The percentage of students who correctly answered TQ5 in the post-test was also only slightly greater than in the pre-test, which was expected given the difficulty of the question. TQ5 was a higher-level Bloom’s Taxonomy question that asked students to apply their understanding of cell–biomaterial interactions to study intervertebral disc degeneration; thus, it would be more difficult for students to execute and implement their knowledge without additional post-activity exercises (e.g., writing a laboratory report).

### Pre/Post-Survey Assessment

**Fig. 6:**
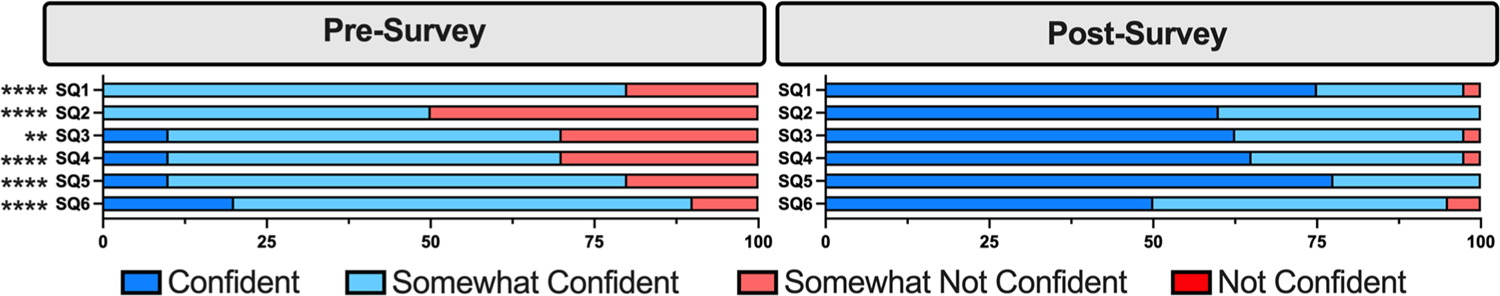
Students showed significant improvements in scientific literacy by pre/post-survey. **p<0.01, ****p<0.0001 using a paired Student’s *t*-test

Validated survey questions (SQs) from the Scientific Literacy and Student Value in Inquiry-guided Lab Survey (SLIGS) were used to assess improvements in scientific literacy (“Appendix 2”) [47]. Pooled surveys of students from all institutions (*N*=40) were analyzed to understand how completing this IBL mechanobiology module impacts students’ scientific literacy. We found that participants felt significantly more confident in all measured metrics of scientific literacy: posing scientific questions (SQ1), assessing methodology (SQ2), providing scientific explanations (SQ3), designing experiments (SQ4), relating scientific discovery to societal impacts (SQ5), and challenging scientific statements (SQ6) (Fig. 6). Therefore, our results demonstrate that completing this IBL mechanobiology module effectively taught students principles of mechanobiology and generally enhanced their confidence with scientific inquiry.

## Discussion

Mechanobiology is important for understanding human disease and developing novel therapeutics [1]. While several educational modules exist to teach students about some topics of mechanobiology [10–18], there remains a need for accessible modules teaching principles of cell–biomaterial interactions. The closest relevant module challenges students to characterize the chemical properties of poly(l-lactic acid) and poly(dl-lactic-*co*-glycolic acid) films, then assess how chemical properties impacted the attachment and proliferation of human dermal fibroblast cells [21]. To the authors’ knowledge, there are no other published modules to teach students about cell–biomaterial interactions, which represents a major gap in the field of mechanobiology. Developing engaging modules to teach principles of mechanobiology to diverse trainees is important because engineering students learn more effectively from these hands-on experiences [49, 50]. Here, we designed, implemented, and evaluated an IBL mechanobiology module to teach undergraduate students principles of cell–biomaterial interactions.

We deployed this IBL mechanobiology module at primarily undergraduate institutions (PUIs) and community colleges to focus our outreach efforts on underrepresented minority (URM) students in science, technology, engineering, and mathematics (STEM). Demographic information collected from individuals who registered for our IBL mechanobiology module confirmed that a vast majority of the students in our cohort identified as URM students. Teaching these demographics of students was important for providing URM students with opportunities to spark their curiosity in STEM. One drawback of our module was that the racial/ethnic backgrounds of the instructors leading the activity did not match those of the students. There is evidence to show that students are more likely to persist in STEM when they are taught by instructors of the same race [51]; thus, assembling diverse instructor teams is important for effectively teaching URM students. To help diversity our team of instructors in a way that mirrored the racial/ethnic background of the students, we recruited staff from the institutions where we deployed the activity to assist in the activity execution. Additionally, we utilized active learning approaches, which diversify the learning experience and help to foster an inclusive classroom environment [52].

When developing this IBL mechanobiology module, we recognized that PUIs and community colleges likely had limited resources, and the populations of URM students who attend these institutions may also come from disadvantaged socioeconomic backgrounds [39]. Therefore, we used agar, *E. coli*, and manual mechanical testing, because these materials are inexpensive and do not require expensive equipment (i.e., mechanical testing devices and biosafety cabinets). Though inexpensive, the hands-on nature of our IBL mechanobiology module was still effective at teaching principles of bioengineering because such hands-on modules help connect theoretical concepts to practical applications [9]. One caveat of this choice is that *E. coli* are prokaryotic cells and not used for regenerative medicine; thus, instructors made a specific point to highlight how the principles learned in the IBL mechanobiology module are applicable to mammalian cell biology. Such innovative and inexpensive outreach modules, which can be easily deployed to URM students in STEM based on race, gender, socioeconomic status, or geographic location [38–40], are important for increasing the number of diverse trainees studying STEM [53, 54]. We recognize that this single module cannot be a panacea for resolving systemic issues around diversity, equity, and inclusion (DEI) in STEM; however, this module is one example of an effective way to empower URM students to advance in STEM.

Pre/post-tests demonstrated that students experienced significant learning gains of 33 percentage points from completing this IBL mechanobiology module. These learning gains were consistent with published bioengineering educational modules that found learning gains of about 50 percentage points for freshman engineering students [46, 55], 15 percentage points for senior engineering students [10], and 15–30 percentage points for classes with mixed undergraduate students [12, 13]. Since our cohort contained students from all undergraduate levels, we expected our learning gains to be within this range. These pre/post-test score increases were primarily driven by larger percentages of students answering TQ1, TQ2, and TQ3 correctly in the post-test. These questions tested concepts of mechanically testing biomaterials, biomaterial synthesis, and cell–biomaterial interactions, which students directly learned though IBL mechanobiology module. Fewer students answered TQ4 correctly in the post-test, which tested transduction of mechanical signals, a concept only brought up in the post-activity exercise. This finding is interesting because TQ1–TQ4 were all similarly low-level questions by Bloom’s Taxonomy [48], designed to have students recall facts. Assessing learning gains with only multiple choice questions represents a slight limitation to this study. Another potential limitation of our approach is that we used the same pre/post-test questions, which could allow students to unconsciously remember questions. To mitigate the latter concern, we did not allow students to review their pre-test answers prior to completing the post-test. Despite these limitations, our findings remain consistent with previous studies demonstrating the enhanced learning of IBL over traditional learning [33, 34].

The IBL mechanobiology module was successful in teaching fundamental principles of mechanobiology; however, additional post-activity exercises are likely required for higher level understanding, as demonstrated by the fact that students performed worst on TQ5, which was the highest level question according to Bloom’s Taxonomy [48]. TQ5 asked students to apply their understanding of cell–biomaterial interactions to study intervertebral disc degeneration. Applications of mechanobiology in studying tissue degeneration and regeneration were discussed in the post-activity exercise; however, students likely required more time with the material and additional post-activity exercises (e.g., writing a lab report) to attain this higher-level thinking. This additional degree of inquiry implementation can help students analyze their data more deeply and draw higher-level conclusions from their results [56]. The module could also be made more complex for a laboratory classroom setting by using more degrees of inquiry implementation. For example, students may begin by making their own observations about cell–biomaterial interactions from the literature, develop broader research questions, design experiments with more independent variables, repeat the experiment after initial failures, and peer review [32]. Many of these additional degrees of inquiry were difficult to implement in our voluntary 2-day module, so we focused our post-activity exercise on discussing limitations of our experiment and applications of mechanobiology. The limitations most frequently raised by students during this post-activity exercise were that our manual mechanical testing scheme was not as accurate as an automated mechanical testing device, and that varying agar percentage changes both stiffness and porosity. In regard to the latter limitation, we explained how the study that motivated our IBL mechanobiology module controlled for the confounding effects of porosity/nutrient transport [45].

Pre/post-surveys showed that students experienced significant improvements in all measured categories of scientific literacy from completing our IBL mechanobiology module. Pre/post-surveys were taken from the Scientific Literacy and Student Value in Inquiry-guided Lab Survey (SLIGS) [47]. We chose this validated survey tool to avoid self-reporting bias, which is a challenge of self-reporting outcome measures [57]. Our findings support the notion that IBL modules effectively teach students the skills to conduct inquiry-based experimentation and raise their confidence with scientific inquiry. This is extremely important for young scientists, especially those who identify as URM, because URM students report having lower confidence in their ability to perform well in STEM coursework [58–60]. Therefore, providing opportunities for URM students to participate in engaging IBL modules, like the one described in this manuscript, can help to combat systemic issues with URM students thriving in STEM. Importantly, scientific literacy is a skill that students must continually develop throughout their scientific careers. To do so, undergraduate students should complete multiple guided modules, Course-based Undergraduate Research Experiences (CURES) [61–63], and eventually transition to independent research projects outside the classroom. In doing so, they will continually develop their scientific literacy skills and prepare themselves for careers as scientific leaders. The IBL mechanobiology module presented is one example of an activity with appropriate technical rigor to improve scientific literacy.

The outlined IBL mechanobiology module was designed to be a 2-day activity; however, instructors may choose to adapt this activity based on their needs. Given the interdisciplinary nature of this IBL mechanobiology module and its effectiveness with a diversity of majors (Fig. 1), we are confident that this module can be utilized in both biology and engineering classrooms. Furthermore, this module could fit into an integrative classroom environment to have biologists and engineers share their strengths with one another [64, 65]. To make the activity more complex for an undergraduate classroom, instructors may have students increase the number of conditions, conduct power analyses to determine appropriate sample sizes, and develop quantitative methods to analyze bacteria growth. These findings should be summarized in a post-activity laboratory report, so students have time to think critically about the material and gain a more advanced understanding of cell–biomaterial interactions in tissue degeneration and tissue regeneration. To reduce the amount of in-class time required for this module, instructors may assign ImageJ analysis as a homework assignment. Additionally, this activity could be shortened to 1 day if instructors do not have students plate their own bacteria, but instead come prepared with bacteria plates that have been incubated overnight.

## Diversity, Equity, and Inclusion

Developing outreach modules that teach relevant principles of science, technology, engineering, and mathematics (STEM) are important for increasing STEM participation [53, 54]. These types of outreach modules are especially important for increasing the representation of underrepresented minority (URM) students in STEM based on race, gender, sexual orientation, socioeconomic status, or geographic location [38–40, 66]. To help address these gaps, we deployed our IBL mechanobiology module at primarily undergraduate institutions (PUIs) and community colleges with high populations of URM students based on race/ethnicity (Fig. 4). We used inexpensive materials and a simple mechanical testing scheme to make our IBL mechanobiology module as accessible as possible to these institutions, which have more limited resources than large research institutions. In addition to teaching URM students about mechanobiology, we also used the module as an opportunity for professional development. Students who completed both days of the module were awarded certificates of completion, provided guidelines of how they could include this module in their CVs, and advised about how they could leverage the skills learned in this module to participate in additional undergraduate research experiences [e.g., the Research Experience for Undergraduates (REU) program offered by our institution]. In addition to targeting these students for our module, we also worked with middle school and high school teachers from Philadelphia to teach them how to incorporate versions of this module into their classrooms. Teachers were encouraged to use Next Generation Science Standards for middle school and high school students to adapt this activity for their classrooms [67, 68]. These modifications would be similar to other published modules, which simplified an undergraduate activity for K-12 outreach [69, 70]. For example, students would be given more guidance when generating hypotheses, conduct less mechanical testing trials, and use simplified statistical methods to analyze the data. We recognize that this single module cannot be a panacea for resolving systemic issues around diversity, equity, and inclusion (DEI) in STEM; however, this module is one example of an effective way to empower URM students to advance in STEM.

## Conclusion

This study designed, implemented, and evaluated a hands-on, IBL mechanobiology module to teach students principles of cell–biomaterial interactions. This hands-on, lowcost, IBL module could be completed in two consecutive days, with 3-h sessions each day. Over the course of the module, students learned how to synthesize and mechanically test biomaterials, culture bacteria cells, and modulate cell phenotype through biophysical stimuli. More broadly, they learned how to apply principles of mechanobiology to study tissue degeneration and promote tissue regeneration. We recruited 40 students, a vast majority of which identified as URM students, to help evaluate our study and found that completing this IBL mechanobiology module resulted in significant learning gains and significant improvements in scientific literacy. Instructors may use this module, as described, to introduce undergraduate students to principles of mechanobiology. Alternatively, we hypothesize that this IBL mechanobiology module could be modified with increased complexity for an undergraduate classroom setting or modified with reduced complexity for K-12 outreach with diverse student populations. Broad deployment of this module, and similar mechanobiology modules, will be useful for encouraging diverse students to study mechanobiology and help addressing the systemic issues surrounding diversity, equity, and inclusion in STEM.

## Figures and Tables

**Table 1:** Inquiry-based learning (IBL) mechanobiology module timeline

Module day	Module stage	Activity section	Duration (min)
Day 1	Pre-activity exercises	Pre-activity test	10
		Instructor introductions	5
		Introduction to mechanobiology	15
	IBL activity	Hypothesis generation and experimental design	10
		Casting agar cubes and *E. coli* plates	70
		Mechanical testing	70
Day 2	IBL activity	ImageJ tutorial	15
		Image and data analysis	90
		Biostatistics tutorial	15
		*E. coli* plate analysis	15
	Post-activity exercises	Post-activity discussion	25
		Post-activity test	10
		Professional development	10

## Data Availability

All test and survey questions can be found in “Appendix 1”. Additional data is available from the corresponding authors upon reasonable request.

## Appendix 1: Pre/Post-Test Questions

1. Synthesizing agar cubes with higher concentrations of agar would:
  - **Increase stiffness by increasing entanglements and crosslinking**
  - Increase stiffness by decreasing entanglements and crosslinking
  - Decrease stiffness by increasing entanglements and crosslinking
  - Decrease stiffness by decreasing entanglements and crosslinking
  - I don’t know
2. Which of the following mechanical tests can determine the compressive stiffness of an agar cube?
  - Cyclically twisting the agar cube
  - Loading the agar cube so that it is extended
  - **Loading the agar cube so that it is shortened**
  - Cyclically loading the agar cube so that it is extended, then shortened
  - I don’t know
3. How will substrate stiffness affect bacterial proliferation?
  - Bacteria will grow faster on stiffer substrates
  - **Bacteria will grow faster of softer substrates**
  - Bacteria growth will be unaffected by substrate stiffness
  - I don’t know
4. Which of the following can change as a result of a cell’s mechanical environment?
  - DNA sequence
  - RNA expression
  - Protein phosphorylation
  - DNA sequence and RNA expression
  - **RNA expression and protein phosphorylation**
  - I don’t know
5. As intervertebral discs (IVDs) degenerate, they undergo degenerative increases in IVD stiffness. You are interested in studying how IVD cells respond to degenerative IVD stiffening. Which experimental setup is best?
  - Culture IVD cells on soft and stiff hydrogels
  - Culture IVD cells with compounds that soften and stiffen their cell membranes
  - **Culture IVD cells on hydrogels with stiffnesses similar to healthy and degenerated IVDs**
  - Culture IVD cells with compounds that soften and stiffen their cell membranes to those of healthy and degenerated IVD cells
  - I don’t know

## Appendix 2: Pre/Post-Survey

Please provide your level of confidence (i.e., Not Confident, Somewhat Not Confident, Somewhat Confident, Confident) with each question of the following prompts below:

1. Post a question that can be addressed through scientific experimentation.
2. Assess the appropriateness of a methodology of an experiment.
3. Provide a scientific explanation for experimental outcomes.
4. Design an experiment that is a valid test of a hypothesis.
5. Relate scientific discovery or idea to the impact on society.
6. Challenge authority on evidence that supports scientific statements.
